# Health-related measures and subjective prognosis of gainful employment among patients with non-specific chronic low back pain in multidisciplinary orthopedic rehabilitation

**DOI:** 10.1055/a-2549-6350

**Published:** 2025-06-10

**Authors:** Petra Hampel, Anne Neumann

**Affiliations:** 1 Dept. Health Psychology and Health Education, Europa-Universität Flensburg, Germany

**Keywords:** subjective prognosis of gainful employment, non-specific chronic low back pain, inpatient multidisciplinary orthopedic rehabilitation, mental health, work-related outcomes, Subjektive Erwerbsprognose, nicht-spezifischer chronischer Rückenschmerz, stationäre verhaltensmedizinisch orthopädische Rehabilitation, psychische Gesundheit, arbeitsbezogene Faktoren

## Abstract

**Purpose:**

Non-specific chronic low back pain (CLBP) restricts participation in society and employment also due to the high psychosocial burden of this condition. Thus, there is an urgent need for rehabilitation of patients with CLBP, which must be determined by a valid diagnosis of psychosocial risk factors. The subjective prognosis of gainful employment (SPE) is considered to indicate the need for medical rehabilitation for back pain. The present study investigated the association between SPE and psychosocial risk factors among individuals with non-specific CLBP undergoing inpatient multidisciplinary orthopedic rehabilitation (MOR).

**Methods:**

This cross-sectional observational study included 925 individuals aged 20 to 65 with non-specific CLBP at admission to inpatient MOR (M=52.2 years, SD=7.2; 77.5% female; ICD-10: M51/53/54). Associations of the SPE total score with psychological, pain-related, and work-related measures were examined by using correlation and regression analyses. Moreover, moderated associations of the SPE categorical score were tested using one-way analyses of variance with the independent factor self-reported prognosis of employment (favorable vs. unfavorable). Additionally, the frequency distributions of scores within the clinical range for depressive symptoms, chronic stress, and subjectively assessed work ability stratified by self-reported prognosis of employment were investigated.

**Results:**

A less favorable self-reported prognosis of employment was predicted by higher job strain and chronic stress as well as lower pain self-efficacy and subjective physical work ability. In particular, individuals with an unfavorable self-reported prognosis of employment showed a risk pattern and were frequently in the clinical range for depressive symptoms, chronic stress, and subjective work ability.

**Conclusion:**

The results supported a high need for rehabilitation for this target group, especially for patients with non-specific CLBP and unfavorable self-reported prognosis of employment. Early assessment of sociomedical criteria, in addition to pain and psychodiagnosis as well as targeted referral to needs-based interdisciplinary multimodal treatment approaches could reduce the risk of further chronification of pain and the development of mental disorders.

## Zusammenfassung

**Hintergrund:**

Nicht-spezifischer chronischer Rückenschmerz (CRS) schränkt auch wegen der hohen psychosozialen Krankheitslast die Teilhabe an Gesellschaft und Arbeitsleben ein. Somit besteht ein dringender Rehabilitationsbedarf für Personen mit CRS, der durch eine valide Diagnostik von psychosozialen Risikofaktoren ermittelt werden muss. Als Indikator für einen Bedarf an einer medizinischen Rehabilitation von Rückenschmerzen wird die subjektive Prognose der Erwerbstätigkeit (SPE) diskutiert. Die vorliegende Studie untersuchte die Zusammenhänge der SPE mit psychosozialen Risikofaktoren bei Personen mit nicht-spezifischem CRS in einer stationären verhaltensmedizinisch orthopädischen Rehabilitation (VMO).

**Methode:**

Die querschnittliche Beobachtungsstudie schloss 925 Personen im Alter zwischen 20 und 65 Jahren mit nicht-spezifischem CRS zu Beginn einer stationären VMO ein (M=52.2 Jahre, SD=7.2; 77.5% weiblich; ICD-10: M51/53/54). Die Zusammenhänge des Gesamtwertes der SPE mit psychologischen sowie schmerz- und arbeitsbezogenen Maßen wurden mittels Korrelations- und Regressionsanalysen untersucht. Außerdem wurden moderierende Zusammenhänge des kategorialen Wertes der SPE anhand einfaktorieller Varianzanalysen mit dem unabhängigen Faktor „Subjektive Erwerbsprognose“ (günstig vs. ungünstig) überprüft. Schließlich wurden die Häufigkeitsverteilungen der klinisch unauffälligen und auffälligen Fälle in der Depressivität und subjektiven Arbeitsfähigkeit sowie im chronischen Stress in Abhängigkeit von der subjektiven Erwerbsprognose untersucht.

**Ergebnisse:**

Eine höhere ungünstige subjektive Erwerbsprognose wurde durch höhere berufliche Belastungen und höheren chronischen Stress sowie eine geringere schmerzspezifische Selbstwirksamkeit und subjektive körperliche Arbeitsfähigkeit vorhergesagt. Insbesondere Personen mit einer ungünstigen subjektiven Erwerbsprognose zeigten ein Risikoprofil und lagen häufiger im klinisch auffälligen Bereich in der Depressivität und subjektiven Arbeitsfähigkeit sowie im chronischen Stress.

**Schlussfolgerung:**

Die Ergebnisse unterstützen, dass ein hoher Rehabilitationsbedarf für diese Zielgruppe besteht, insbesondere für Personen mit nicht-spezifischem CRS und einer ungünstigen subjektiven Erwerbsprognose. Eine frühzeitige Erfassung von sozialmedizinischen Kriterien zusätzlich zu der Schmerz- und Psychodiagnostik und eine gezielte Zuweisung zu bedarfsgerechten interdisziplinären multimodalen Behandlungsangeboten könnte das Risiko einer weiteren Chronifizierung der Schmerzen und die Entwicklung psychischer Störungen mindern.

## Introduction

In 2017, low back pain (LBP) became the third most prevalent condition in Western Europe, with an age-standardized point prevalence of 13% being the leading cause of years lived with disability (YLDs) compared to all other conditions in the Global Burden of Disease Study 1 . Globally, the age-standardized point prevalence of LBP increased with age, but YLDs were highest in the working-age population aged 45 to 49. In 2020, LBP ranked also first worldwide in YLDs, mainly attributed to occupational ergonomic factors, and a significant increase in YLDs related to LBP was predicted for 2050 2 . Moreover, a high risk for the transition from acute to chronic LBP (CLBP) has been reported and, finally, most CLBP cannot be explained by specific pathological causes and has to be classified as non-specific CLBP 3 . In epidemiological and clinical studies, significant predictors of chronicity have been found 4 . Overall, not only psychosocial factors but also work- and workplace-related factors have been shown to increase both the risk of a chronic course and the maintenance of CLBP 3 4 . Psychosocial factors, especially depressive symptoms, somatization, as well as maladaptive pain-related cognitions and behavioral pain coping were associated with LBP disability (for a review, see 3 ; for somatization, see e. g., 5 ). Additionally, pain self-efficacy is increasingly of interest because of its strong association with pain disability 3 and its mediating effects on the relationship between pain and disability 6 . Similarly, pain self-efficacy mediated the longitudinal relationship between depressive symptoms and work-related outcomes among patients with CLBP 7 . Work dissatisfaction 3 and negative self-reported prognosis of return-to-work (RTW) have been found to be the most significant work-related risk factors 8 . Finally, co-existing mental symptoms have been frequently reported to exacerbate chronicity; 19.5% of patients with CLBP were diagnosed with at least one comorbid mental disorder 9 . A recent study found that patients with comorbid somatic and mental disorders had similar psychological impairments compared to patients with only mental disorders 10 . Therefore, co-existing mental symptoms enhance not only the psychological burden but also the socioeconomic burden, resulting in enhanced frequencies of disability pensions 9 .

Hence, early identification of vulnerable subgroups of people with back pain (BP) is highly important for providing them with access to rehabilitation programs that are tailored to their needs and intended to prevent the exacerbation of pain and to promote participation in society and employment. Early detection of the need for rehabilitation and systematic treatment allocation are even more important since a recent cohort study among employees with BP showed that only 25.6% of individuals with a potential need for rehabilitation had an intention of applying for rehabilitation 11 . Further research among people with BP and musculoskeletal disorders supported that the self-reported prognosis of employment may be a significant predictor of a need for rehabilitation, disability pension, and interrupted employment 12 , for applying for a pension and taking early retirement 13 , and failure to RTW (cf., 14 ). Moreover, self-reported prognosis of employment, assessed by the Subjective Prognosis of gainful Employment Scale (SPE; 15 ), showed cross-sectional associations with pain-related measures as well as some psychological and work-related measures, using administrative data 11 or data collected in an intervention study prior to an inpatient orthopedic rehabilitation (OR) 14 . In the 2–3 year longitudinal study among 481 blue-collar workers with severe BP or functional complaints in the area of internal medicine provided by Mittag et al. 13 , the predictive value of the SPE for employment status was the same or even higher than that for other predictors of employment, such as work dissatisfaction, comorbid disorders, and depressive symptoms. Thus, Bethge et al. 14 already mentioned in 2007, that an unfavorable self-reported prognosis of employment in the OR is considered an important indicator of specific work-related problems.

However, previous results on the association of SPE among people with BP and musculoskeletal disorders were mainly restricted to administrative data 11 12 13 , collected in a mostly male sample 13 14 of blue-collar workers 13 with lower percentages of an extremely unfavorable self-reported prognosis of employment of approximately 6% 16 , or focused on sociomedical variables 12 13 . Hence, the aim of the present cross-sectional study was to extend these earlier findings to the context of German inpatient multidisciplinary orthopedic rehabilitation (MOR) for non-specific CLBP. MOR is part of behavioral medicine-oriented rehabilitation, which has been established in Germany over the last 20 years by the German Pension Insurance (DRV) to provide patients with somatic disorders and psychological comorbidities with standardized psychological diagnostics and psychological or psychotherapeutic co-treatment in somatic rehabilitation (e. g., 17 ). Patients with BP in MOR are therefore characterized by multiple psychological strains and an increased risk for the development, maintenance, or exacerbation of non-specific CLBP. Therefore, MORs are differentiated from ORs, which are targeted to people suffering from orthopedic conditions without mental strain. The present study also aimed to extend the findings regarding the associations of SPE to further well-established psychological (pain self-efficacy, chronic stress), work-related (subjective work ability, job strain), and pain-related variables (pain sites).

Overall, an unfavorable SPE was expected to be linked to a worse health status. In addition, it was expected that patients with an unfavorable self-reported prognosis of employment would more frequently achieve normative scores in the clinical range for depressive symptoms, chronic stress, and subjective work ability. Finally, regarding the association of the SPE total score with health-related measures among patients with non-specific CLBP in the MOR, stronger associations with work ability and work-related stress were expected, but rather weaker associations with psychological variables. The weak empirical evidence on the pain-related parameters suggests that pain disability correlates rather highly with the SPE total score 11 , while pain intensity correlates rather moderately 11 14 .

## Methods

### Study design and procedure

The present secondary analysis of the registered, controlled trial Debora 7 18 19 was based on a cross-sectional observational design. Data were collected within the prospective multicenter study Debora 7 18 19 from October 2014 to December 2015 in four German inpatient MOR clinics at rehabilitation onset. While the physician determined the stage of pain during an initial consultation, the self-assessment questionnaires were completed prior to the rehabilitation, which occurred on the premises of the cooperating clinics in the presence of documentation staff.

All participants were informed about the study aims during their initial physical consultation at the clinic and each provided written informed consent. This study was approved by the ethical review board of the German Psychological Society (DGPs) and was conducted in accordance with the 1964 Helsinki Declaration and its later amendments. Moreover, the trial was retrospectively registered with the German Clinical Trials Registry in 2018 (DRKS00015465).

### Participants

All participants were consecutively included if they were aged between 20 and 65 prior to rehabilitation, had a diagnosis of CLBP lasting at least 6 months (ICD-10: M51, M53, M54), provided informed consent for participation, and were fluent in German. The exclusion criteria for the study were a specific etiology of CLBP, accidents/surgeries within the last 6 months, severe psychological and somatic comorbidities, and pregnancy.

### Variables

#### Psychological variables

*Depressive symptoms* were determined by the sum scores of the 20 4-point Likert items of the German version of the Center for Epidemiological Studies Depression Scale (CES-D; 20 ). The internal consistency of the CES-D estimated by Cronbach’s alpha was 0.93. Principal component factor analysis (PCA) revealed for the 1-factor solution that the factor explained 39% of the total variance. This measure seemed to be suitable for screening depressive symptoms in the context of MOR 21 . Moreover, the well-established *Short Screening Scale for Chronic Stress* (SSCS) of the Trier Inventory for Chronic Stress was measured (TICS; cf., 22 23 ). This short version consisted of 12 5-point rating items and incorporated items from the following subtests: chronic worrying, work overload, social overload, excessive demands at work, and lack of social recognition. The Cronbach’s alpha was 0.91, and PCA showed for the 1-factor solution that the factor explained 50% of the total variance. Patient confidence in their ability to perform several activities despite pain, or *pain self-efficacy* , was assessed by the 10-item German version of the Pain Self-Efficacy Questionnaire (PSEQ; 24 ). The Cronbach’s alpha was 0.93, and PCA supported that the scale was unidimensional, accounting for 66% of the total variance. The two physical and mental health subtests of the Short-Form 12 (SF-12) measured *health-related quality of life* with 6 items each 25 . The Cronbach’s alpha values for physical and mental health were 0.91 and 0.92, respectively, and PCAs supported that the subtests were unidimensional, accounting for 53% and 54% of the total variance, respectively. Higher scores indicated better quality of life (0–100).

#### Work-related variables

The *self-reported prognosis of employment* was assessed using the SPE, which consisted of the following 3 items ( 15 ; for translation, cf., 11 ):

- When you think about your current state of health and your ability to work, do you think you will be able to work until you reach retirement age (certainly, rather yes, uncertain, rather no, in no case)?
- Do you see your current state of health as a permanent endangerment of your employability (no, yes)?
- Are you currently considering applying for a pension (disability pension) (no, yes)?

The first 5-point item had to be dichotomized first, summarizing the first two and the last three scores. All three binary items were summed to the SPE total score, ranging from 0 to 3. Therefore, higher scores of the SPE total score indicated an unfavorable SPE. The Cronbach’s alpha was 0.68, and PCA supported that the scale was unidimensional, explaining 61% of the total variance. Additionally, a categorical score was suggested, with scores of “0” and “1” as a favorable SPE but scores of “2” and “3” as an unfavorable SPE 11 13 . Two single 5-point Likert items from the Work Ability Index (WAI) were used to assess subjective physical and mental *work ability* (cf., 26 ). The WAI ranged from 7 to 49, with higher scores indicating higher work ability. The 3 5-point rating items of the scale *job strain* were taken from the Würzburg Screening 27 . The Cronbach’s alpha was 0.79, and PCA supported that the scale was unidimensional, explaining 71% of the total variance.

Pain-related variablesThe perceived functional disability in everyday activities due to BP was assessed by 12 3-point rating items of the Hannover Functional Ability Questionnaire for measuring BP-related functional limitations (FFbH-R; 28 ). The internal consistency of the *functional capacity* was measured with a Cronbach’s alpha 0.93. The PCA revealed for the 1-factor solution that the factor explained 42% of the total variance. The scores ranged from 0 to 100%. *Pain disability related to work* and the *average pain intensity* were estimated on an 11-point rating scale taken from the German Questionnaire of Pain (DSF; 29 ). Pain disability was evaluated in relation to the last 3 months, and pain intensity was evaluated as deviation from the DSF in relation to an observation period of two weeks. In addition, the current number of *pain sites* was taken from the DSF. Based on the sum score, the 3 *stages of pain* of the German Mainz Pain Staging System (MPSS; 30 ) were determined 30 . To classify the *pain grading* in the four groups, 7 items were taken from the DSF 29 and calculated in accordance with the chronic pain grade index 31 .

### Statistical analysis

Relationships were examined for the SPE total score by first determining the associations with the other variables through a bivariate approach using Spearman’s rank correlation and point-serial correlation, respectively, with pairwise exclusion. Furthermore, the correlation was also analyzed taking into account potential covariates using multiple linear regression analysis (cf., 15 ), with listwise exclusion. The following potential covariates were included (cf., 14 ): gender, age, social status, average pain intensity, pain sites, pain staging, pain grading, and job strain. In the first step, all possible predictors were included in an exploratory multiple linear regression analysis. Only the predictors with p<0.05 were used in a second step in a backward multiple linear regression analysis (cf., 14 ).

Previous studies indicate adequate sensitivity and specificity of the SPE with its cut-off score at > 1 13 . A current study provided by Fauser et al. 12 , supported this finding and concluded: “An SPE score of at least 2 points was therefore defined as a threshold to screen people at risk (p. 91 f.)“. Thus, in the present study, associations were also investigated for the SPE categorical score, differentiating between favorable and unfavorable SPE.

Two procedures were applied: a) In line with 11 14 , group differences in psychological, work-related, and pain-related variables were first parametrically tested using multi- and univariate one-way analyses of variance (ANOVAs). The between-subjects factor represented the self-reported prognosis of employment, which was categorized into two groups: favorable vs. unfavorable. For this purpose, the SPE total score was dichotomized (0–1 vs. 2–3; 11 13 ).

To reduce the amount of multiple testing, ANOVA was preferred to Student’s *t* tests. Both subtests on quality of life and both items of the WAI were analyzed by multivariate ANOVAs with subsequent univariate ANOVAs to localize the effects of the MANOVA. Additional Mann-Whitney *U* tests were performed to confirm the ANOVA results to control for unequal sample sizes in both experimental groups. If the different methods yielded different effect sizes, the smaller value was used for interpretation.

b) In order to obtain further information on the clinical relevance of the SPE categorical score, the associations were also examined using standardized measures for depressive symptoms, chronic stress, and subjective work ability. The frequency distributions of individuals with clinical and subclinical scores were examined by χ ^2^ tests stratified by self-reported prognosis of employment (favorable vs. unfavorable). For this purpose, the measures were dichotomized beforehand: For depressive symptoms, the recommended cut-off score of > 22 was applied. For the screening scale of chronic stress, *T* values above 60 were considered to be clinical. Finally, for dichotomization, the WAI was classified as clinical with poor extent (7–27) or subclinical with moderate, good, or very good extent (28–49).

In addition to the eta-square, Cohen’s *d* was calculated as a further effect size for the tests for group differences to provide comparability in general (cf., 32 ) and with previous relevant studies 11 14 . Omega-square values are not reported here. Overall, these values were 0.001 lower than eta-square and the effect sizes did not change substantially. However, due to the explorative nature of this study, the significance level was set at *p* <0.050 without applying a Bonferroni correction. The effect sizes were interpreted as small, moderate, or large: η ^2^ :≥0.010,≥0.059, or≥0.138; Cohen’s *d* ≥0.20,≥0.50, or≥0.80; β, Cramer’s *V* , ρ or *r* ≥0.10,≥0.30, or≥0.50; and *R* ^2^ ≥0.02,≥0.13, or≥0.26 33 . Moreover, confidence intervals ( *CIs* ) were calculated for eta-square, Cohen’s *d* and the unstandardized coefficient *B* .

## Results

### Recruitment and participants

A total of 2075 patients were asked to participate in the study; 769 patients did not agree to participate (response rate: 62.94%). Of these 1306 individuals, 381 were excluded who had missing data at the baseline assessment in the sum scores of the German version of the CES-D 20 , the German version of the PSEQ 24 , the SPE 15 , and in single items of pain intensity and pain sites of the DSF 29 , in the pain staging (MPSS; 30 ), in the pain grading 31 , and in gender. Furthermore, some participants were excluded because of evidence of response bias in the CES-D.

The 381 excluded individuals differed from the 925 included individuals in only two measures; they were more likely to be employed at least half a week (χ² _(7)_ =14.21, *p* =0.048, Cramer’s *V* =0.11) and reported more than 14 days of sick leave in the past 3 months (χ² _(1)_ = 4.38, *p* =0.036, Cramer’s *V* =0.06). For differences in age, gender, family status, and pain duration, the result was p>0.05.

In total, *n* =925 individuals were included in the analyses. They had a mean age of 52.15 years ( *SD* =7.19), were mostly female (77.5%, *n* =717), and were employed (87.9%, *n* =780). According to the social class index 34 , the majority belonged to the middle class (55.2%, *n* =463), which is composed of the 3 individual indicators school-leaving qualification, occupational status, and net household income. Slightly more than half had a critical value for subjective work ability (50.8%, *n* =440). The mean pain duration was 14.06 years ( *SD* =10.47). Apart from gender, age, marital status, and pain duration, patients with an unfavorable self-reported prognosis of employment had significantly worse social, work-related, and pain-related scores ( **Suppl Table 1** ).

### Association between the SPE total score and health-related measures

Spearman’s rank correlation or point-serial correlation revealed that the SPE total score was not associated with the potential covariates gender or age (p≥0.05) and was correlated with the other potential covariates, although mostly with small effect sizes (p<0.05; **Suppl Table 2** ). For the possible somatic predictors “physical health”, “functional capacity”, and “pain disability related to work”, correlations tended to show moderate effect sizes. In addition, large effect sizes were found for pain self-efficacy, job strain, and physical work ability. Finally, psychological predictors tended to be moderately related or related with small effect sizes (mental health) to the SPE total score. An increased SPE total score was associated with unfavorable levels of physical, mental, and social well-being.

In the exploratory multiple linear regression analysis with the SPE total score as the dependent variable, a total variance of 41% could be explained. Five significant predictors were identified ( Table 1 ). In the backward multiple linear regression analysis, the final model with these remaining predictors explained 40% of the total variance, but had small effect sizes only for job strain, chronic stress, pain self-efficacy, and physical work ability ( *F* (5,631)=84.08, *p<* 0.001; Table 2 ): Enhanced job strain and chronic stress as well as decreased pain self-efficacy and physical work ability predicted an increased SPE total score.

**Table:** **Table 1** Results of the multiple linear regression analysis of the SPE total score (initial model; *n* =637).

					95%- *CI (B)*	
	β	*B*	*SE (B)*	*p*	*Lower CI*	*Upper CI*
**(Constant)**		1.653	0.733	0.024	0.214	3.092
**Gender** (male)	0.039	0.097	0.080	0.225	− 0.060	0.255
**Age**	− 0.049	− 0.008	0.005	0.126	− 0.017	0.002
**Social status** (3=upper class)	− 0.043	− 0.071	0.053	0.182	− 0.175	0.033
**Days of sick leave** (>2 weeks)	0.021	0.047	0.086	0.587	− 0.122	0.215
**Average pain intensity (DSF)**	− 0.037	− 0.020	0.022	0.346	− 0.063	0.022
**Pain sites (DSF)**	0.073	0.032	0.015	0.036	0.002	0.062
**Pain staging** (stage III)	0.018	0.028	0.051	0.589	− 0.073	0.129
**Pain grading** (grade IV)	0.000	0.000	0.047	0.997	− 0.092	0.093
**Job strain (Würzburg Screening)**	0.188	0.077	0.018	<0.001	0.042	0.112
**Depressive symptoms (CES-D)**	0.022	0.002	0.005	0.679	− 0.008	0.012
**Screening scale for chronic stress (TICS)**	0.097	0.013	0.006	0.027	0.001	0.025
**Pain self-efficacy (PSEQ)**	− 0.185	− 0.017	0.004	<0.001	− 0.025	− 0.009
**Physical health (SF-12)**	− 0.025	− 0.003	0.006	0.634	− 0.016	0.010
**Mental health (SF-12)**	0.041	0.004	0.005	0.446	− 0.006	0.014
**Physical work ability (WAI)**	− 0.209	− 0.226	0.050	<0.001	− 0.325	− 0.128
**Mental work ability (WAI)**	− 0.080	− 0.089	0.047	0.057	− 0.182	0.003
**Functional capacity (FFbH-R)**	− 0.074	− 0.004	0.002	0.091	− 0.009	0.001
**Pain disability related to work (DSF)**	− 0.034	− 0.012	0.020	0.541	− 0.052	0.027
***R*** ^**2**^	0.414					
**Adjusted** ***R*** ^**2**^	0.397					

DSF=German Questionnaire of Pain. CES-D=Center for Epidemiological Studies Depression Scale. TICS=Trier Inventory for Chronic Stress. PSEQ=Pain Self-Efficacy Questionnaire. SF-12=Short Form-12. WAI=Work Ability Index. FFbH-R=Hannover Functional Ability Questionnaire – back pain. *CI* =Confidence interval. *B* =Unstandardized coefficient. β=Standardized coefficient. *SE* =Standard error.

**Table:** **Table 2** Results of the multiple linear regression analysis of the SPE total score (final model; *n=* 637).

					95%- *CI (B)*	
	β	*B*	*SE (B)*	*p*	*Lower CI*	*Upper CI*
**(Constant)**		0.737	0.365	<0.044	0.020	1.455
**Pain sites (DSF)**	0.078	0.034	0.014	0.016	0.006	0.062
**Job strain (Würzburg Screening)**	0.216	0.089	0.017	<0.001	0.056	0.122
**Screening scale for chronic stress (TICS)**	0.107	0.014	0.005	0.004	0.005	0.024
**Pain self-efficacy (PSEQ)**	− 0.220	− 0.020	0.003	<0.001	− 0.027	− 0.014
**Physical work ability (WAI)**	− 0.242	− 0.261	0.043	<0.001	− 0.346	− 0.177
***R*** ^**2**^	0.400					
**Adjusted** ***R*** ^**2**^	0.395					

DSF=German Questionnaire of Pain. TICS=Trier Inventory for Chronic Stress. PSEQ=Pain Self-Efficacy Questionnaire. WAI=Work Ability Index. CI=Confidence interval. B=Unstandardized coefficient. β=Standardized coefficient. SE=Standard error.

### Association between the SPE categorical score and health-related measures

MANOVA results revealed high effect sizes for health-related quality of life and subjective work ability ( Table 3 ). Overall, in the non-parametric analyses, associations with p<0.05 found in the parametric analyses could be confirmed, but showed some lower effect sizes. Mann-Whitney *U* Tests and the one-way ANOVAs revealed differences in all measures between individuals with favorable vs. unfavorable SPE (p<0.05; Table 3 , **Suppl Table 2** ). The large effect sizes for pain self-efficacy, physical health, work ability, job strain, and functional capacity in the parametric analyses were confirmed non-parametrically by only moderate effect sizes. Both analyses resulted in moderate effect sizes for chronic stress, mental work ability, and pain disability related to work. The moderate effect size for depressive symptoms could only be validated by a small effect size non-parametrically. Consistently, parametric and non-parametric analyses yielded small effect sizes for mental health, pain sites, and average pain intensity. Individuals with an unfavorable SPE showed psychological, work-related, and pain-related impairments.

**Table:** **Table 3** Means ( *M* ) and standard errors ( *SE* ) and ANOVA results of the SPE categorical score ( *n=* 925).

Variable		Prognosis of employment							
		Favorable ( *n* =489)	Unfavorable ( *n* =436)		*F* -statistics				
				*df(1,2)*	*F*	η ^2^	*95%-CI(* η ^*2*^ *)*	*d*	95%- *CI* ( *d* )
**CES-D**	***M***	20.38	26.96						
	***SE***	0.48	0.51	1.923	087.04	0.086	0.06− 0.12	− 0.614	− 0.75- − 0.48
**TICS**	***M***	21.07	26.53						
	***SE***	0.35	0.38	1.905	111.85	0.110	0.08− 0.15	− 0.704	− 0.84- − 0.57
**PSEQ**	***M***	42.29	32.38						
	***SE***	0.48	0.51	1.923	201.88	0.179	0.14− 0.22	0.936	0.80–1.07
**Physical health (SF-12)** ^1^	***M***	39.84	32.78						
	***SE***	0.37	0.39	1.923	170.27	0.156	0.12− 0.20	0.859	0.72− 0.99
**Mental health (SF-12)** ^1^	***M***	40.53	35.69						
	***SE***	0.48	0.51	1.923	047.64	0.049	0.03− 0.08	0.455	0.32− 0.59
**Physical WAI** ^2, 3^	***M***	3.14	2.24						
	***SE***	0.04	0.04	1.913	249.56	0.212	0.17− 0.26	1.038	0.90–1.18
**Mental WAI** ^2, 3^	***M***	3.15	2.49						
	***SE***	0.04	0.04	1.913	121.62	0.117	0.08− 0.16	0.728	0.59− 0.86
**Job strain (Würzburg Screening)** ^4^	***M***	10.11	12.36						
	***SE***	0.11	0.11	1.914	217.05	0.192	0.15− 0.24	− 0.975	−1.11- − 0.84
**Functional capacity (FFbH-R)**	***M***	71.92	56.51						
	***SE***	0.82	0.87	1.923	166.09	0.153	0.11− 0.19	0.849	0.71− 0.98
**Pain sites (DSF)**	***M***	4.70	5.86						
	***SE***	0.11	0.12	1.923	052.34	0.054	0.03− 0.08	− 0.477	− 0.61- − 0.35
**Pain intensity (DSF)**	***M***	4.63	5.49						
	***SE***	0.09	0.09	1.923	048.00	0.049	0.03− 0.08	− 0.456	− 0.59- − 0.33
**Pain disability (DSF)** ^5^	***M***	4.39	6.50						
	***SE***	0.12	0.13	1.919	144.65	0.136	0.10− 0.18	− 0.794	− 0.93- − 0.66

*df* =degrees of freedom. η ^2=^ eta-square and Cohens *d* ( *effect sizes* ). *CI* =Confidence interval. CES-D=Center for Epidemiological Studies Depression Scale. TICS=Trier Inventory for Chronic Stress. PSEQ=Pain Self-Efficacy Questionnaire. SF-12=Short Form-12. WAI=Work Ability Index. FFbH-R=Hannover Functional Ability Questionnaire – back pain. DSF=German Questionnaire of Pain. *p’s<* 0.001; ^1^ MANOVA _SF-12_ *F*_(6,2010)_ =89.74, *p<0.* 001, *η²=0.* 211). ^2^ MANOVA _WAI_ *F*_(6,1984)_ =60.33, *p<0.* 001, *η²=0.* 154). ^3^ *N* =915. ^4^ *N* =916. ^5^ *N* =921.

Testing the frequency distributions of clinical and subclinical scores for the selected parameters confirmed most of the findings of the analyses of group differences ( Table 4 ). Patients with a favorable SPE showed higher frequencies of scores in the subclinical range, as expected, and patients with an unfavorable SPE showed higher frequencies of scores in the clinical range, as expected. Here, a large effect size was found for the dichotomized WAI. In contrast, small effect sizes were found for depressive symptoms and chronic stress.

**Table:** **Table 4** Observed and expected frequencies of the SPE categorical score.

		Subjective prognosis of employment		
		Favorable ( *n* =489)	Unfavorable ( *n* =436)	∑ ( *n* =925)
**CES-D – Depressive symptoms** (χ² _ (1, *n* = 925)= _ 50.85, *p* <0.001, *V* =0.234)				
Subclinical (total score≤22)	Observed (%)	292 (31.6)	158 (17.1)	450 (48.6)
	Expected	238	212	450
Clinical (total score>22)	Observed (%)	197 (21.3)	278 (30.1)	475 (51.4)
	Expected	251	224	475
**TICS – Screening scale for chronic stress** (χ² _ (1, *n* = 907) **=**_ 69.04, *p* <0.001, *V* =0.276)				
Subclinical (T≤60)	Observed (%)	309 (34.1)	155 (17.1)	464 (51.2)
	Expected	247	217	464
Clinical (T>60)	Observed (%)	173 (35.9)	270 (29.8)	443 (48.8)
	Expected	235	208	443
**WAI – Work Ability Index** (χ² _ (1, *n* = 866)= _ 238.60, *p* <0.001, *V* =0.525)				
Subclinical (total score≥28)	Observed (%)	343 (39.6)	83 (9.6)	426 (49.2)
	Expected	230	196	426
Clinical (total score<28)	Observed (%)	124 (14.3)	316 (36.5)	440 (50.8)
	Expected	237	203	440

CES-D=Center for Epidemiological Studies Depression Scale. TICS=Trier Inventory for Chronic Stress. WAI=Work Ability Index. χ²=Chi-square. *V* =Cramer´s *V* ( *effect size* ).

## Discussion

In summary, in line with previous findings from studies among people with BP and musculoskeletal disorders 11 14 , patients with non-specific CLBP in the MOR with an unfavorable self-reported prognosis of employment were characterized by impaired health-related measures.

As expected, the results of all correlational analyses and analyses for group differences showed a close association between the SPE and the single item on *physical work ability* and the *WAI* . Here, 36.5% of the total sample had an unfavorable self-reported prognosis of employment and a critical value in the WAI. Another single item of the WAI, the work ability score, has itself recently been shown to be a predictor of health-related early retirement and work disability among people with BP 16 . Overall, these findings among individuals with BP or musculoskeletal disorders support the assumption of Fauser et al. 12 that SPE is a valid indicator of work disability 12 . However, further results from a 5-year longitudinal study with a representative sample size of 4.225 statutory pension insurance members collected administrative data and found insufficient positive predictive values 35 . Although its predictive value is therefore limited, it is still recommended that SPE be considered in a comprehensive test battery to screen for vulnerable patient subgroups. It has been shown that such screening-based decisions on access to rehabilitation measures have enabled more needs-oriented access to work-related medical rehabilitation 36 .

Moreover, a close relationship between SPE and the subtest *job strain* was confirmed by all analyses. Consistently, previous cross-sectional analyses found significant predictions of SPE for job performance even with a small effect size 15 and job strain 14 . In addition, the *SSCS* showed moderate effect sizes in the bivariate correlations and group comparisons but predicted the SPE total score with a small effect size in the final regression model. The SSCS measures chronic stress more in relation to work, which explains the high intercorrelation with the subtest job strain in the present sample ( *r* =0.526, *p* <0.001, *N* =898). Likewise, a significant prediction has already been demonstrated for job satisfaction and work-related striving for perfection 14 , again underscoring the importance of work-related stress and its coping for an unfavorable self-reported prognosis of employment among individuals with BP. In sum, the increased job strain and the high proportion of a critical WAI of 50.8% in the present sample reflect the increased strain due to complex specific work-related problems and imply that stress management training should be explicitly incorporated into psychological treatments in the MOR for non-specific CLBP.

Inconsistent findings emerged regarding to *pain-related parameters* . Group differences stratified by the SPE categorical score with a high effect size for pain disability 11 and a moderate effect size for pain intensity were confirmed 11 14 . However, pain intensity proved to be a significant predictor in the initial and final models of the multiple logistic regression analysis with the SPE categorical score as a criterion variable 14 , which could not be confirmed here. In addition, functional capacity predicted the SPE total score with a small effect size in multiple linear regression analysis 15 , which was also not confirmed. In the present analysis, however, an impact of the number of pain sites was found. For this, a small effect size was shown bivariately, and this parameter proved to be a significant predictor in the initial regression model. Thus, the findings suggest that the number of pain sites is important for an unfavorable self-reported prognosis of employment among patients with non-specific CLBP in MOR. In line with previous studies, the number of pain sites was related to low health status and high symptom burden at both the physical and mental levels (cf., 37 ) and has been shown in multivariable predictive models together with pain intensity and psychological distress to be predictors of the risk of developing disabling LBP 3 . Thus, future studies should examine the relationship between pain intensity or pain sites and pain disability mediated by SPE.

In contrast, *psychological parameters* such as depressive symptoms and mental health were less strongly associated with the SPE, which has already been shown for the subtests vitality and well-being of the SF-36 15 . The moderate effect for depressive symptoms could only be confirmed as a small effect size in the non-parametric analyses of group differences. In contrast, a prior cross-sectional analysis showed a large effect size in unpaired Student’s *t* tests but used a different measurement tool and examined a younger and less affected sample with only 27% of people having an unfavorable self-reported prognosis of employment 11 . Likewise, the findings of the frequency distributions of clinical scores of the CES-D confirmed small effect sizes. However, 30% of the total sample showed both an unfavorable self-reported prognosis of employment and clinical scores for depressive symptoms. Again, the results reflect the psychosocial risk profile of this sample and suggest that depression prevention training should be integrated into MOR programs for non-specific CLBP as addressed by Debora (cf., 19 38 ).

For the first time, the association with the cognitive variable *pain self-efficacy* was tested here and proved to be large in the bivariate analyses and small in the final model of the multiple linear regression analysis. The lower association of SPE with depressive symptoms but the stronger relationship with pain self-efficacy may be attributed to the mediating effects of pain self-efficacy. According to a previous analysis by Debora, high pain self-efficacy 12 months after rehabilitation predicts decreased levels of the SPE total score and increased work ability 24 months after rehabilitation, and pain self-efficacy mediates the long-term prediction of work-related factors by depressive symptoms 7 . Thus, it is suggested that increased pain self-efficacy is an essential psychological protective factor for the development of an unfavorable self-reported prognosis of employment among individuals with non-specific CLBP.

Overall, it can be concluded that an unfavorable self-reported prognosis of employment was accompanied by impaired psychological, pain-related, and work-related scores. These results support that both SPE and psychological measures should be included in a comprehensive screening approach when allocating vulnerable patient subgroups to tailored rehabilitation interventions according to their needs (cf., 14 ). Due to the low level of evidence for the influence of pain intensity at the start of multidisciplinary biopsychosocial treatment on rehabilitation success, Kamper et al. 39 already recommended that physical and psychosocial risk factors should be taken into account. However, the long-term rehabilitation effects were rather small and receding. In own studies, favorable mid- and long-term effects on the WAI 18 19 , and mid-term effects on pain-related days of sick leave 19 were found after a German inpatient MOR for non-specific CLBP, in which psychological treatment elements were implemented to promote pain self-efficacy. However, no incremental long-term effects of Debora were found for depressive symptoms or anxiety 18 40 . Similarly, incremental effects were confirmed for the work ability score, self-management skills, pain disability, and fear-avoidance beliefs 10 months after inpatient MOR compared to medical rehabilitation, but no effects were found on RTW, applications for disability pension, and the number of patients receiving social security benefits 41 . Future studies are needed to investigate whether the extension of sociomedical and pain-specific assessments by the SPE and psychological parameters followed by early systematic allocation to needs-based interdisciplinary multimodal rehabilitation can prevent under-, mis- and overtreatment and improve the effectiveness of interventions.

Limitations  Despite replicating previous findings on the association of SPE with health-related measures among people with BP and musculoskeletal disorders, some limitations must be discussed. First, due to filter variables, 381 patients were excluded from the analyses. However, this ensured that only cases with complete data were included in the analysis. Dropout analyses revealed a significant difference only for employment status, with a small effect size between patients who dropped out and patients who stayed in the study. Thus, systematic bias is not assumed. Second, the results are restricted by the sample characteristics of this study: Among the patients with non-specific CLBP who were allocated to MOR according to specific criteria, most were female, which is in agreement with the study provided by Markus et al. 41 . Previous population-based studies have shown that women have a higher prevalence of CLBP in Germany 42 43 and worldwide 44 . Moreover, it has been shown that females are more at risk for developing chronic pain, which might be mediated by higher pain catastrophizing 45 . However, gender and the SPE categorial score was not associated in the present study. In addition, this sample had a longer pain history with higher pain grades; 16% of the sample had an extremely unfavorable self-reported prognosis of employment and half had clinical scores indicating depressive symptoms and chronic stress. In contrast to earlier studies, which included individuals with less pain intensity, lower pain grades, and a more restricted age range (e. g., 45–59; 11 12 ). Additionally, half of this sample belonged to the middle class. Previous studies have provided evidence for a significant impact of social inequity, showing higher pain intensity, multisite pain, and mental impairments and, in another analysis of this data set, no beneficial rehabilitation effects among patients of the lower class 18 . Therefore, in this study, the prediction of the SPE total score was controlled for social status, but no essential confounding effect was suggested. Third, although a representative sample was investigated in a multicenter study, the results are based on a cross-sectional observational design. Thus, causal assumptions cannot be drawn. However, longitudinal data have supported the prognostic validity of the SPE 12 13 . Finally, although well-established measures were used, administrative data were not available, and the results are limited to self-reports. However, emotional and cognitive processes represent covert processes, whose assessment by proxy-reports tends to be invalid 46 .

## Key message

Earlier findings of the associations of SPE with health-related measures were replicated and extended to a) the context of German inpatient MOR for non-specific CLBP and b) further psychological, work-related, and pain-related variables. Patients with an unfavorable self-reported prognosis of employment were characterized by a risk pattern in work-related and pain-related measures and mental health status. Thus, early identification of this vulnerable subgroup through a comprehensive sociomedical, pain-related, work-related, and psychological test battery and systematic allocation to tailored multidisciplinary rehabilitation may prevent the development of chronic pain and co-existing mental disorders.

## Funding

This work was supported by the Deutsche Rentenversicherung Bund (German Pension Insurance Central) under grant 8011–106–31/31.115.
