# Supplementary material for: Health-related measures and subjective prognosis of gainful employment among patients with non-specific chronic low back pain in multidisciplinary orthopedic rehabilitation
Source: Rehabilitation (Stuttg). 2025 Jun 10;64(3):146–56. doi: 10.1055/a-2549-6350 (PMC12151593; doi:10.1055/a-2549-6350)
Supplement: Supplementary file 1 — Supplementary Material [file 10-1055-a-2549-6350-2024-05-0027.pdf]

Supplementary Material

Suppl Table 1 Participant characteristics for subjective prognosis of gainful employment (SPE).

Variable	SPE favorable (*n* = 489)	SPE unfavorable (*n* = 436)	total (*n* = 925)	*n*	*p*-value
**Socio-demographic data**					
Age [in years] [a] *Mean ± SD*	52.53 ± 6.81	51.72 ± 7.57	52.15 ± 7.19	925	$p > .05$
Gender, female [b] *no.* (%)	389 (42.1)	328 (35.5)	717 (77.5)	925	$p > .05$
Family status (married) [b] *no.* (%)	294 (32.4)	262 (28.9)	556 (61.2)	908	$p > .05$
Social status [b] *no. (%)*				839	$p < .001$
lower class	51 (06.1)	87 (10.4)	138 (16.4)		
middle class	247 (29.4)	216 (25.7)	463 (55.2)		
upper class	148 (17.6)	90 (10.7)	238 (28.4)		
**Work-related data**					
Employed [b] *no.* (%)	445 (50.2)	335 (37.8)	780 (87.9)	887	$p < .001$
Pain-related DSL [b] ≤ 2 weeks, *no.* (%)	295 (40.3)	162 (22.1)	457 (62.4)	732	$p < .001$
Work Ability Index [b], *Total score, no.* (%)				866	$p < .001$
poor	124 (14.3)	316 (36.5)	440 (50.8)		
moderate	268 (30.9)	78 (09.0)	346 (40.0)		
good	71 (08.2)	5 (00.6)	76 (08.8)		
excellent	4 (00.5)	0 (00.0)	4 (00.5)		
**Pain history**					
Pain duration [in years] [b] *Mean ± SD*	13.89 ± 10.65	14.26 ± 10.26	14.06 ± 10.47	836	$p > .05$
Pain staging [b] *no.* (%)				925	$p < .001$
I	159 (17.2)	80 (08.6)	239 (25.8)		
II	240 (25.9)	216 (23.4)	456 (49.3)		
III	90 (09.7)	140 (15.1)	230 (24.9)		
Pain grading [b] *no.* (%)				925	$p < .001$
I	118 (12.8)	38 (04.1)	156 (16.9)		
II	83 (09.0)	33 (03.6)	116 (12.5)		
III	166 (17.9)	129 (13.9)	295 (31.9)		
IV	122 (13.2)	236 (25.5)	358 (38.7)		

SD = Standard deviation. [a] analyses of variance. [b] chi2 test. DSL = days of sick leave.

Suppl Table 2 Rank correlations of the SPE total score.

Variable	*r**	*p*	*n*
**Gender** (1=female, 2=male)	.035	.289	925
**Age**	-.026	.435	925
**Social status** (1=lower, 2=middle, 3=upper class)	-.204	<.001	839
**Days of sick leave** (1=≤ 2 weeks, 2= > 2 weeks)	.323	<.001	732
**Average pain intensity (DSF)**	.269	<.001	925
**Pain sites (DSF)**	.259	<.001	925
**Pain staging** (1=stage I, 2=stage II, 3=stage III)	.229	<.001	925
**Pain grading** (1=grade I, 2=grade II, 3=grade III, 4=grade IV)	.369	<.001	925
**Job strain (Würzburg Screening)**	.533	<.001	916
**Depressive symptoms (CES-D)**	.352	<.001	925
**Screening scale for chronic stress (TICS)**	.366	<.001	907
**Pain-related self-efficacy (PSEQ)**	-.501	<.001	925
**Physical health (SF-12)**	-.451	<.001	925
**Mental health (SF-12)**	-.263	<.001	925
**Physical work ability (WAI)**	-.558	<.001	916
**Mental work ability (WAI)**	-.400	<.001	918
**Functional capacity (FFbH-R)**	-.450	<.001	925
**Pain disability related to work (DSF)**	.407	<.001	921

r = Spearman's rank correlation coefficient for continuous and ordinal variables and point-biserial correlation for dichotomous variables. DSF = German Questionnaire of Pain. CES-D = Center for Epidemiological Studies Depression Scale. TICS = Trier Inventory for Chronic Stress. PSEQ = Pain Self-Efficacy Questionnaire. SF-12 = Short Form-12. WAI = Work Ability Index. FFbH-R = Hannover Functional Ability Questionnaire – back pain.

Suppl Table 3 Median (*MD*) and mean rank (*rank*) and Mann-Whitney *U* results of the SPE categorical score (n = 925).

		Prognosis of employment				
**Variable**		**Favorable (*n*=489)**	**Unfavorable (*n*=436)**	***U*-statistics**		
**Depressive symptoms (CES-D)**	***MD***	19.00	27.66	***Z***	***p***	***V***
	Rank	389.99	544.89	*-08.81*	< .001	.290
	n	489	436			
**Screening scale for chronic stress (TICS)**	***MD***	21.00	27.00	***Z***	***p***	***V***
	Rank	373.60	545.18	*-09.85*	< .001	.327
	n	482	425			
**Pain self-efficacy (PSEQ)**	***MD***	43.00	31.00	***Z***	***p***	***V***
	Rank	569.45	343.61	*-12.84*	< .001	.422
	n	489	436			
**Physical health (SF-12)**	***MD***	38.79	32.25	***Z***	***p***	***V***
	Rank	563.18	350.64	*-12.08*	< .001	.400
	n	489	436			
**Mental health (SF-12)**	***MD***	38.70	34.34	***Z***	***p***	***V***
	Rank	519.53	399.60	*-06.82*	< .001	.224
	n	489	436			
**Physical work ability (WAI)**[1]	***MD***	03.00	02.00	***Z***	***p***	***V***
	Rank	568.72	333.93	*-14.11*	< .001	.467
	n	486	430			
**Mental work ability (WAI)**[1]	***MD***	03.00	03.00	***Z***	***p***	***V***
	Rank	540.21	368.70	*-10.30*	< .001	.340
	n	486	432			
**Job strain (Würzburg Screening)**[2]	***MD***	10.00	13.00	***Z***	***p***	***V***
	Rank	345.08	584.47	*-13.78*	< .001	.456
	n	482	434			
**Functional capacity (FFbH-R)**	***MD***	75.00	56.25	***Z***	***p***	***V***
	Rank	561.39	352.65	*-11.89*	< .001	.391
	n	489	436			
**Pain sites (DSF)**	***MD***	04.00	05.00	***Z***	***p***	***V***
	Rank	407.52	525.22	*-06.75*	< .001	.222
	n	489	436			
**Average pain intensity (DSF)**	***MD***	05.00	05.00	***Z***	***p***	***V***
	Rank	408.96	523.61	*-06.60*	< .001	.217
	n	489	436			
**Pain disability related to work (DSF)**[3]	***MD***	04.00	07.00	***Z***	***p***	***V***
	Rank	369.40	563.79	*-11.13*	< .001	.366
	n	487	434			

Z = Z-score. *V* = Cramer's *V* (effect size). CES-D = Center for Epidemiological Studies Depression Scale. TICS = Trier Inventory for Chronic Stress. PSEQ = Pain Self-Efficacy Questionnaire. SF-12 = Short Form-12. WAI = Work Ability Index. FFbH-R = Hannover Functional Ability Questionnaire – back pain. DSF = German Questionnaire of Pain. [1]*N*=915. [2]*N*=916. [3]*N*=921.
